# Supplementary material for: The genome sequence of the Beautiful Golden Y, Autographa pulchrina (Haworth, 1809)
Source: Wellcome Open Res. 2023 Aug 30;8:375. [Version 1] doi: 10.12688/wellcomeopenres.19840.1 (PMC11739698; doi:10.12688/wellcomeopenres.19840.1)
Supplement: Peer review history

# Reviewer report: Reviewer response for version 1

**DOI:** 10.21956/wellcomeopenres.21972.r88454

**Published:** 2025-01-16

**Authors:** Marko Mutanen

**Affiliations:** 1 University of Oulu, Oulu, Finland

This is a well-prepared paper of a widely distributed and well-known species of *Plusiinae*. The genome is very useful for example to address the taxonomic problems in *Plusiinae* and the genus *Autographa*. I find it unfortunate that the authors compare this species with A. jota, which is a clearly distinct species, rather than its true sister A. *buraetica* that is present in the mainland Europe and could be easily overlooked. It is also so similar to *pulchrina *that their taxonomic status as separate species have sometimes been questioned. Also, because of this similarity, and possible overlook of buraetica, it would have been important to confirm that the studies specimen truly represents pulchrina. Based on the voucher photograph, this appears likely, but that this aspect is completely ignored is an issue. That buraetica perhaps is not known to occur in British Isles is not an excuse in cases like this. The DNA barcodes appear slightly, but constantly different.

- In the Introduction, its last paragraph, the species name is misspelled once: *A. pulichrina* > *A. pulchrina*.
- That the larvae feed on "low plants" is a bit misleading as I find its larvae typically in woody bushes such as willows. They themselves mention honeysuckle. I do not find its typical food plants "low plants" while they probably are used too.
- As a general comment that concerns many genome reports is that the species have been introduced like if they were occurring in British Isles only. I would appreciate a bit wider geographic focus with these reports.
- The genome part appears very strong as all the statistics of genome quality suggest so. For example, the BUSCO percentage is very high and well over the benchmark. K-mer completeness value is also very high.

Are sufficient details of methods and materials provided to allow replication by others?

Yes

Is the rationale for creating the dataset(s) clearly described?

Yes

Are the datasets clearly presented in a useable and accessible format?

Yes

Are the protocols appropriate and is the work technically sound?

Yes

Reviewer Expertise:

Molecular taxonomy, phylogenetics, Lepidoptera, Sawflies, DNA barcoding, biodiversity genomics, dark taxa

I confirm that I have read this submission and believe that I have an appropriate level of expertise to confirm that it is of an acceptable scientific standard.

# Reviewer report: Reviewer response for version 1

**DOI:** 10.21956/wellcomeopenres.21972.r66279

**Published:** 2023-10-20

**Authors:** Reza Zahiri

**Affiliations:** 1 Entomology, State Museum of Natural History Karlsruhe, Karlsruhe, Baden-Württemberg, Germany

Review of ''The genome sequence of the Beautiful Golden Y, *Autographa pulchrina''*

 by Douglas Boyes and Kaouthar Eljounaidi

 This is a well-written paper, very straightforward and transparent from the Wellcome Centre and Darwin Tree of Life project. There's nothing much to report except a few minor comments can be found below:

 Abstract

 The species name should come with the author's name as appears for the first time in the paper.

 How if you modify it this way:

 ... an individual female the Beautiful Golden Y ( *Autographa pulchrina *(Haworth, 1809): Arthropoda; Insecta; ...

 Keywords

 Usually, keywords are meant to be different from the title, e.g., use Plusiinae, Noctuoidea, genome assembly

 Background

 Line 1. ... member of the family Noctuidae.

 Line 2. of the order Lepidoptera, ...

 Line 3. this paper should be cited as well:

https:/www.mapress.com/zt/article/view/zootaxa.3148.1.41

 Line 7: Plain Golden Y ( *Autographa jota*) needs the author's name

 Line 8: by close comparison of the forewings - and probably genitalic characteristics?

 Last paragraph. The genome of *A. pulichrina* was sequenced as part -> The genome of *A. pulichrina* is sequenced as part 

 Last line. dynamics of the Lepidoptera order -> dynamics of the order Lepidoptera

 Genome sequence report

 Line 1. how is this specimen collected?

 Methods

 Para. 1. ... and preserved on dry ice in a CoolRack. -> without being killed by a killing agent, if so please indicate.

Are sufficient details of methods and materials provided to allow replication by others?

Yes

Is the rationale for creating the dataset(s) clearly described?

Yes

Are the datasets clearly presented in a useable and accessible format?

Yes

Are the protocols appropriate and is the work technically sound?

Yes

Reviewer Expertise:

Evolutionary biologist

I confirm that I have read this submission and believe that I have an appropriate level of expertise to confirm that it is of an acceptable scientific standard.

## References

[1] : Order Lepidoptera Linnaeus, 1758. In: Zhang, Z.-Q. (Ed.) Animal biodiversity: An outline of higher-level classification and survey of taxonomic richness. *Zootaxa* .2011;3148(1) : 10.11646/zootaxa.3148.1.41 10.11646/zootaxa.3148.1.41 26146682
